# In Vitro Antioxidant Activity of Liposomal Formulations of Sea Buckthorn and Grape Pomace

**DOI:** 10.3390/foods13162478

**Published:** 2024-08-07

**Authors:** Violina Popovici, Adrian-Bogdan Boldianu, Adela Pintea, Vladimir Caraus, Aliona Ghendov-Mosanu, Iurie Subotin, Raisa Druta, Rodica Sturza

**Affiliations:** 1 Faculty of Food Technology, Technical University of Moldova, 9/9 Studentilor St., MD-2045 Chisinau, Moldova; aliona.mosanu@tpa.utm.md (A.G.-M.); iurie.subotin@fta.utm.md (I.S.); raisa.druta@fta.utm.md (R.D.); rodica.sturza@chim.utm.md (R.S.); 2 Faculty of Veterinary Medicine, University of Agricultural Sciences and Veterinary Medicine, 400374 Cluj-Napoca, Romania; adrian-bogdan.boldianu@student.usamvcluj.ro (A.-B.B.); apintea@usamvcluj.ro (A.P.); 3 CSK Grup Plus SRL, MD-2021 Chisinau, Moldova; caraus.vadim1965@gmail.com

**Keywords:** carotenoids, polyphenols, liposomes, encapsulation, bioactive compounds, compound stability

## Abstract

This study evaluated the impact of the encapsulation of sea buckthorn and grape pomace extracts in liposomal formulations on the retention and release of bioactive compounds and their antioxidant activity. The profile and composition of lipophilic extracts of sea buckthorn and hydrophilic extracts of grape pomace were analyzed. Encapsulation efficiency, retention rate, and the content of bioactive compounds encapsulated in liposomal formulations prepared in two media—water and ethanol—were evaluated. The encapsulation efficiency varied between 84 and 90%, indicating the superior encapsulation of the bioactive compounds. The retention rate varied between 79 and 86%, which indicated the stability of the liposome-encapsulated compounds over time. The antioxidant activity of the encapsulated samples was determined in vitro, under the conditions of gastric (pH 1.8) and intestinal (pH 8.2) digestion, in relation to the non-encapsulated extracts. The antioxidant activity of both liposomal formulations was higher than that of the nonencapsulated extracts during gastric digestion. Moreover, an increase over time in the antioxidant activity, expressed as % DPPH inhibition, was observed for all samples, with around 90% DPPH inhibition for non-encapsulated extracts and 92% for the encapsulated extracts, demonstrating the stability of bioactive compounds in acidic pH. Oppositely, when exposed to intestinal simulated digestion (alkaline pH), the antioxidant activity decreased over time to around 24% DPPH inhibition for both encapsulated and nonencapsulated extracts. These results provide a foundation for the further development and application of liposomal delivery systems in functional foods.

## 1. Introduction

Food safety is a notable concern for consumers and the food industry. A series of complex challenges associated with food engineering and food industries, including the production of quality food and food safety through efficient and feasible means, can be overcome through nanotechnology. Liposome nanoparticles are regarded as attractive new materials by the food and pharmaceutical industries [1]. For example, the nanoencapsulation of bioactive compounds is an emerging application of nanotechnology. In various industrial food practices, liposome nanoparticles have been used to enhance the flavor and nutritional properties of foods and examined for their ability to encapsulate natural metabolites that can help protect foods from spoilage and degradation [2,3].

Oxidation of biomolecules causes serious health issues such as cancer, cardiovascular diseases, cataracts, and diabetes [4]. A possible strategy to protect against oxidation is to use antioxidant compounds. The application of natural antioxidants instead of synthetic ones is increasing due to the deleterious effects of synthetic antioxidants, including the possibility of carcinogenesis and liver damage [5,6]. The application of plant-based extracts as natural antioxidants is vital to protect various food products against oxidation [7,8]. Among the most important classes of natural antioxidants are polyphenolic compounds, commonly used to prevent oxidation and increase the shelf life of food material [9,10]. The protection of bioactive components, including vitamins, antioxidants, fatty acids, phytosterols, lycopene, lutein, minerals, and live probiotic cells, is essential for most food and health applications, both within food systems and in their efficient delivery to the human body. Both carotenoids and polyphenols are sensitive compounds. Therefore, various factors such as oxygen level, pH, temperature, and light can induce structural changes and reduce their antioxidant properties [11]. These compounds can be entrapped in a carrier system to increase their stability while protecting them against degradation and oxidation during storage and maintaining their antioxidant activity [12,13].

Liposomal encapsulation is a promising technique for enhancing the stability and bioavailability of bioactive compounds. One of the primary advantages of liposomal encapsulation is the enhanced stability of bioactive compounds. Liposomal formulation protects polyphenols and carotenoids from oxidative degradation, thereby extending their shelf life and maintaining their biological activity. This protection is crucial for preserving the health benefits of these compounds during storage, processing, and consumption. For instance, liposome-encapsulated resveratrol, a polyphenol found in grapes, has shown significantly improved stability and bioavailability compared to its free form [14,15].

Another significant advantage is the improved bioavailability of liposome-encapsulated compounds. Bioavailability refers to the proportion of a nutrient or bioactive compound that reaches the systemic circulation and becomes available for physiological functions. Many polyphenols and carotenoids have low bioavailability due to their poor solubility in water and/or rapid metabolism in the gastrointestinal tract [16]. Liposomal formulations enhance the solubility of these compounds, facilitate their absorption through the intestinal lining, and protect them from enzymatic degradation [17].

The encapsulation of polyphenols and carotenoids in liposomes offers a versatile and effective approach to overcoming the limitations associated with these bioactive compounds [18,19]. By enhancing their stability, bioavailability, and targeted delivery, liposomal formulations can maximize the health benefits of polyphenols and carotenoids, paving the way for their broad application in functional foods, dietary supplements, and pharmaceutical products. As research in this field continues to advance, liposomal encapsulation can play a crucial role in the development of next-generation nutraceuticals and therapeutics [13].

This study aimed to evaluate the impact of the encapsulation of sea buckthorn lipophilic extract rich in carotenoids and red grape pomace extract rich in polyphenols in liposomal formulations on the retention and release of bioactive compounds and the antioxidant activity of these compounds.

## 2. Materials and Methods

### 2.1. Materials and Chemicals

Sea buckthorn fruits (*Hippophae rhamnoides* L.) of the “Clara” variety were harvested from a plantation in Pohrebea village (Republic of Moldova) [20]. Red grape pomace (*Vitis vinifera* L.) of the “Merlot” variety was obtained from the Pilot Laboratory of Microvinification at the Technical University of Moldova, Chisinau, Republic of Moldova). Sea buckthorn fruit and grape pomace were dried at 65 ± 1 °C to a moisture content of 6.8 ± 0.4%, grounded to a powder, and sieved. Refined and deodorized sunflower oil “Floris” (Balti, Republic of Moldova) was used to obtain the lipophilic extracts. Ethanol solution (60%, *v*/*v*) was used to obtain hydrophilic extracts. Egg yolk phospholipids (DPPC) were purchased from LIPOID GmbH (Ludwigshafen, Germany). Acetonitrile of HPLC purity was purchased from Merck (Darmstadt, Germany) and ultrapure water was purified using the Direct-Q UV system from Millipore (Burlington, MA, USA). Chlorogenic acid, gallic acid, rutin, cyanidin, and catechin (purity > 99% HPLC) were sourced from Sigma (Burlington, MA, USA). Carotenoid standards (β-carotene, lycopene, β-cryptoxanthin, lutein, zeaxanthin, and zeaxanthin dipalmitate) were purchased from Extrasynthese (Genay, France). All other chemicals used were of analytical grade.

### 2.2. Extract Preparation

For the polyphenol extract of grape pomace (PE), the grape pomace powder was mixed with a 60% vol. aqueous ethanol solution at a ratio of 1:10, with the extraction of bioactive compounds performed by stirring at 1000 rpm at 45 ± 1 °C for 120 min. The solution was then filtered and the aqueous ethanol solution was removed by evaporation using a rotary evaporator, resulting in a polyphenol extract with a dry matter content of 40%; the extract was stored at a temperature of 4 ± 1 °C [21].

For the carotenoid-enriched extract from sea buckthorn (LE), the sea buckthorn powder was obtained by convective drying of the raw material (45 ± 1 °C) to a moisture content of 7%, grinding (70 µm), mixing with refined and deodorized sunflower oil at a ratio of 1:10, the extraction of bioactive compounds by ultrasonication (37 kHz) at 45 ± 1 °C for 180 min, and separation of the lipophilic extract by centrifugation followed by filtration; the extract was stored at a temperature of 4 ± 1 °C and protected from light [22]. All extractions were performed in triplicate.

### 2.3. Analysis of Phenolic Compounds in Grape Pomace

The extract was dissolved in the mobile phase and filtered through a Chromafil Xtra nylon 0.45 µm filter, and 20 μL was injected into the HPLC system. For the separation and quantification of phenolic compounds, the Agilent 1200 HPLC system was used, equipped with a quaternary pump, solvent degasser, autosampler, and UV–Vis photodiode array detector (DAD) and coupled with an Agilent 6110 single quadrupole mass detector (MS) (Agilent Technologies, Santa Clara, CA, USA). The separation of phenolic compounds was performed on a Kinetex XB C18 column with dimensions of 4.6 × 150 mm and with 5 μm particles (Phenomenex, Torrance, CA, USA) using the mobile phases (A) water + 0.1% acetic acid and (B) acetonitrile + 0.1% acetic acid with the gradient below over 30 min at a temperature of 25 °C and a flow rate of 0.5 mL/min. Gradient (expressed in % B): 0 min, 5% B; 0–2 min, 5% B; 2–18 min, 5–40% B; 18–20 min, 40–90% B; 20–24 min, 90% B; 24–25 min, 90–5% B; 25–30 min, 5% B. Spectral values were recorded in the range of 200–600 nm for all peaks. Chromatograms were recorded at wavelengths λ = 280, 340, and 520 nm. Data acquisition and interpretation of the results were performed using Agilent ChemStation software, version B.02.01 SR2. The phenolic compounds were identified by comparing the retention time, UV visible, and mass spectra data with those of the available standards, literature data, and Phenol-Explorer database. Positively charged ions were detected by mass spectrometry, using the Scan mode. The following conditions for mass spectrometry were used: gas temperature 350 °C, nitrogen flow 7 L/min, nebulizer pressure 35 psi, capillary voltage 3000 V, fragmentor 100 V, and *m*/*z* 120–1200. Phenolic compounds were quantified using five-point calibration curves built with gallic acid, catechin, chlorogenic acid, rutin, and cyanidin [23].

### 2.4. Analysis of Carotenoids in Sea Buckthorn Samples

The extract was dissolved in the mobile phase and the resulting solution was then subjected to HPLC analysis after filtration with 0.22 μm filter paper. Carotenoids were separated on a YMC C30 reversed-phase column (250 × 4.6 mm i.d., 5 µm particle size) using an LC20 AT HPLC system (Shimadzu Corporation, Kyoto, Japan) equipped with an SPDM20A diode array detector. A gradient elution was applied, with the mobile phase consisting of methanol/tert-butyl methyl ether/water (83:15:2, *v*/*v*/*v*) (solvent A) and tert-butyl methyl ether/methanol/water (80:7:2, *v*/*v*/*v*) (solvent B) at a flow of 0.8 mL/min. The gradient program was as follows: 0 min 0% solvent B, 20 min 0% B; 130 min 82% B; 132 min 0% B, followed by equilibration of the column for 10 min. The chromatograms were recorded in the range of 300–600 nm for the acquisition of UV–Vis spectra, while the chromatograms for quantitative analysis were extracted at 450 nm. The identification of carotenoids was performed by comparing their retention times and UV–Vis spectra (including spectral fine structure) with those of the available standards and literature data. Quantitative analysis was performed using external calibration with β-carotene, lycopene, lutein, zeaxanthin, and zeaxanthin dipalmitate in the range of 1–100 µg/mL [24].

### 2.5. Free Radical Scavenging Method (DPPH)

To determine antioxidant activity, the method described by Brand-Williams et al. [25] was used. To a 0.1 mM solution of DPPH˙ in MeOH (3.9 mL), an amount of sample (0.1 mL) was added, and the reaction mixture was shaken vigorously by hand. The reduction of DPPH˙ absorbance was followed by monitoring at 517 nm after 30 min. As a control, the absorbance of a blank solution of DPPH˙ (3.9 mL) was also registered at 517 nm. The results were expressed as % DPPH [26].

### 2.6. Liposome Preparation

Encapsulated bioactive-compound liposomes were prepared by an adapted heating method (Mozafari method) described by Rasti et al. [27]. Briefly, the mixture of the liposomal ingredients, including egg yolk PLs (preheated to 30 °C) and bioactive extracts of polyphenols or carotenoids (2:0.4, mass ratio), were hydrated by adding distilled water or ethanol solution and glycerol (final concentration 2%, *v*/*v*) (preheated to 30 ± 1 °C) and stirred at 1000 rpm on a hotplate (VELP Scientifica, ARE-6, Usmate Velate, Italy) at 30 °C for 60 min. The preparation process was carried out in a glass volumetric flask with sonication for 4 min in a bath-type sonicator in the continuous mode at 37 °C to reduce liposome size. We prepared liposomal formulations for each media: liposomal formulation with carotenoids in double-distilled water (CDW); liposomal formulation with polyphenols in double-distilled water (PDW); liposomal formulation with carotenoids in ethanol (CEt); and liposomal formulation with polyphenols in ethanol (PEt). The liposomal samples were stored at 4 ± 1 °C after preparation until further tests.

### 2.7. Encapsulation Efficiency

The determination of encapsulation efficiency (EE) was determined by extracting bioactive compounds to measure the free amounts of carotenoids and polyphenols in the suspension; aliquots of 0.5 mL loaded liposomes were mixed with 3 mL of ethanol and stirred using a vortexer for 3 min at room temperature. The mixture was then centrifuged at 2000 rpm for 5 min to collect the supernatant. This procedure was repeated twice. The combined supernatants were diluted to a final volume of 10 mL with ethanol. The free bioactive compounds were quantified spectrophotometrically using a UV–Vis spectrophotometer (UV-1800, Shimadzu, Kyoto, Japan) in triplicate [9,28]. The EE was calculated using the following equation:

(1) $$EE\;\left(\%\right)=\frac{total\;amount\;of\;bioactive\;compounds-free\;bioactive\;compound}{total\;amount\;of\;bioactive\;compounds}\times 10$$

### 2.8. Retention Rate

To evaluate the shelf life of the liposomes, the samples were stored at 4 ± 1 °C and, at specific time intervals, an aliquot from the sample was removed to measure the retention rate (RR). The measurement of leaked bioactive compounds during storage was performed in the same way as described above to obtain the free amount of carotenoids and, respectively, of polyphenols [28]. The RR was quantified as follows:

(2) $$RR\;\left(\%\right)=\frac{encapsulated\;amount\;of\;bioactive\;compounds\;after\;storage}{encapsulated\;amount\;of\;carotenoid\;initially\;prepared}\times 100$$

### 2.9. In Vitro Antioxidant Activity

The determination of in vitro antioxidant activity was carried out in 2 phases, which consisted of the gastric phase and the intestinal phase. For the gastric stage, samples of 10 g of the product were dissolved in 100 mL of HCl solution with pH = 1.8 ± 0.1 and 15 mg of pepsin was added. For the intestinal stage, similar samples of 10 g of the product were dissolved in 100 mL of NaHCO_3_ solution with pH = 8.2 ± 0.1 and 15 mg of trypsin was added. The samples were thermostated at 37.0 ± 0.1 °C by continuous stirring at 95 rpm for 120 min. From the initial samples (time 0) as well as after 60 min and 120 min, aliquots were taken for the spectrophotometric determination of the antioxidant activity using a DPPH assay. Each aliquot was centrifuged for 10 min (6000 rpm) and then the filtrate (1 mL each) was subjected to analysis. The determination of the antioxidant activity of the aliquots was performed by the direct reaction: 3.9 mL of DPPH solution and 0.1 mL of the analyzed sample. Methanol was used as a reference sample. The reaction took place for 30 min in a dark place. The absorbance at 515 nm was then read on the “SHIMADZU-UV-1800” spectrophotometer [29].

### 2.10. Statistical Analysis

The calculations were executed using Microsoft Office Excel 2007 (Microsoft, Redmond, WA, USA). The data obtained in this study are presented as mean values ± standard error of the mean, calculated from triplicate experiments. Statistical analysis was performed using analysis of variance (ANOVA) with Tukey’s post hoc test, with a significance threshold of *p* ≤ 0.05. The Statgraphics Centurion XVI version 16.1.17 software (Statgraphics Technologies, Inc., The Plains, VA, USA) was employed for these analyses.

## 3. Results

### 3.1. Characterization of Extracts

The carotenoid content in the pulp of sea buckthorn (*Hippophae rhamnoides* L.) was evaluated in a non-saponified state, and the results are summarized in Table 1.

The analysis of the carotenoid content in the non-saponified pulp of sea buckthorn *(Hippophae rhamnoides* L.) provided a detailed insight into its nutritional composition. The quantified carotenoids highlight the potential health benefits of sea buckthorn pulp, emphasizing its role as a valuable dietary source of bioactive compounds. 

**Figure 1:**
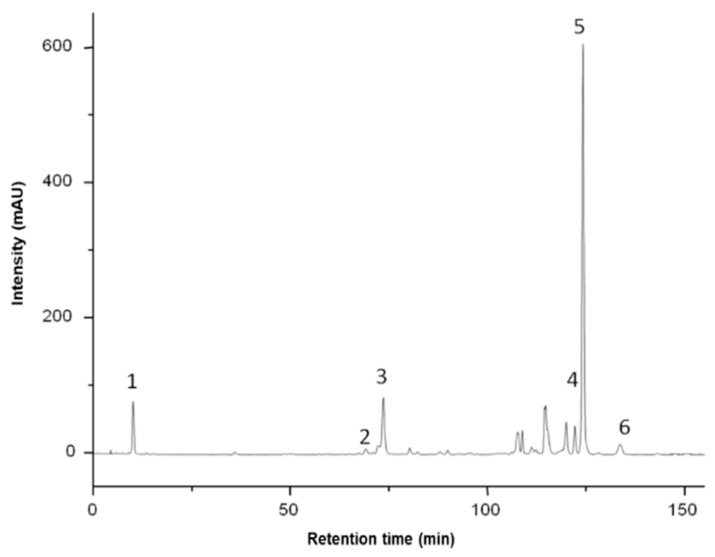
Non-saponified sea buckthorn pulp extract chromatogram: (1) zeaxanthin; (2) cis-*β*-carotene; (3) all-*trans-β*-carotene; (4) lutein dipalmitate; (5) zeaxanthin dipalmitate; (6) zeaxanthin palmitate-stearate.

The concentration of zeaxanthin in the non-saponified sea buckthorn pulp was found to be 0.72 mg/100 g FW (Table 1, Figure 1). Previous studies conducted by Ghendov-Mosanu et al. showed that zeaxanthin content in saponified sea buckthorn pulp increased to 2.54 mg/100 g FW [20]. This increase can be attributed to the hydrolysis of zeaxanthin esters (such as zeaxanthin dipalmitate and zeaxanthin palmitate-stearate) into free zeaxanthin.

Significant amounts of all-*trans*-*β*-carotene (0.79 mg/100 g FW) and small amounts of cis-*β*-carotene (0.20 mg/100 g FW), zeaxanthin dipalmitate (4.53 mg/100 g FW), zeaxanthin palmitate-stearate (0.47 mg/100 g FW), and lycopene (0.14 mg/100 g FW) were found in the sea buckthorn extract. 

The analysis of the grape pomace extract revealed a diverse and rich profile of phenolic compounds, as summarized in Table 2. These compounds were distributed across several subclasses, including hydroxybenzoic acids, flavanols, anthocyanins, flavonols, and hydroxycinnamic acids. The phenolic compounds chromatograms are shown in the Supplementary Materials (Figures S1–S3).

The presence of 3-hydroxybenzoic acid (7.23 mg/100 g DW) and 2-hydroxybenzoic acid (7.08 mg/100 g DW) indicated a substantial content of simple phenolic acids. Gallic acid-gallate (9.66 mg/100 g DW), another significant hydroxybenzoic acid, highlighted the contribution of this subclass to the total phenolic content.

Among the flavonols, quercetin derivatives were noteworthy. Quercetin-glucoside (3.24 mg/100 g DW), quercetin-glucuronide (3.63 mg/100 g DW), and quercetin-acetyl-glucoside (1.07 mg/100 g DW) illustrated the complex glycosylation patterns of this flavonol in the extract. Kaempferol-rutinoside (1.05 mg/100 g DW), kaempferol-acetyl-glucoside (2.10 mg/100 g DW), and isorhamnetin-glucoside (2.88 mg/100 g DW) also contributed to the flavonol content, indicating a variety of glycosylated kaempferol derivatives.

Flavanols dominated the phenolic profile, with significant amounts of catechin (20.47 mg/100 g DW) and epicatechin (47.67 mg/100 g DW). The high concentration of these monomeric flavanols suggests potent antioxidant activity and potential health benefits. Additionally, procyanidin dimers, including prodelphinidin dimer B9 (8.72 mg/100 g DW), procyanidin dimer B3 (16.66 mg/100 g DW), procyanidin dimer B1 (11.80 mg/100 g DW), and procyanidin dimer B2 (18.39 mg/100 g DW), highlighted the presence of oligomeric flavanols, which are known for their strong antioxidative properties and cardioprotective effects.

Anthocyanins were well represented in the grape pomace extract, though at lower concentrations compared to flavanols [14]. Malvidin derivatives were particularly notable, with malvidin-glucoside (0.84 mg/100 g DW), malvidin-glucoside-pyruvic acid (1.06 mg/100 g DW), malvidin-acetyl-glucoside (0.98 mg/100 g DW), malvidin-caffeoyl-glucoside (1.41 mg/100 g DW), and malvidin-p-coumaroyl-glucoside (0.90 mg/100 g DW) present. Other anthocyanins, such as peonidin-glucoside (0.47 mg/100 g DW) and petunidin-*p*-coumaroyl-glucoside (1.65 mg/100 g DW), contributed to the color and antioxidant properties of the extract.

Hydroxycinnamic acids, represented by caffeic acid-glucoside (5.69 mg/100 g DW) and 3,4-dicaffeoylquinic acid (4.55 mg/100 g DW), were also significant contributors. These compounds are known for their antioxidant activity and potential role in protecting against various diseases.

The extensive phenolic profile of the grape pomace extract demonstrates its potential as a source of diverse bioactive compounds. The high concentrations of flavanols, particularly catechin and epicatechin, suggest that the extract has strong antioxidant properties, which can be beneficial for several preventive medicine applications, such as reducing oxidative stress and improving cardiovascular health. The presence of various glycosylated flavonols and anthocyanins adds complexity to the extract, potentially enhancing its bioavailability and efficacy in biological systems [30,31].

Moreover, the notable quantities of hydroxybenzoic and hydroxycinnamic acids contribute to the overall antioxidant capacity of the extract, making it a potentially valuable component for functional foods, nutraceuticals, and cosmetic formulations. The diverse phenolic content underlines the value of grape pomace as a resource for extracting bioactive compounds that can be utilized in various industries for health-promoting products.

### 3.2. Preparation and Characterization of Liposomes

**Figure 2:**
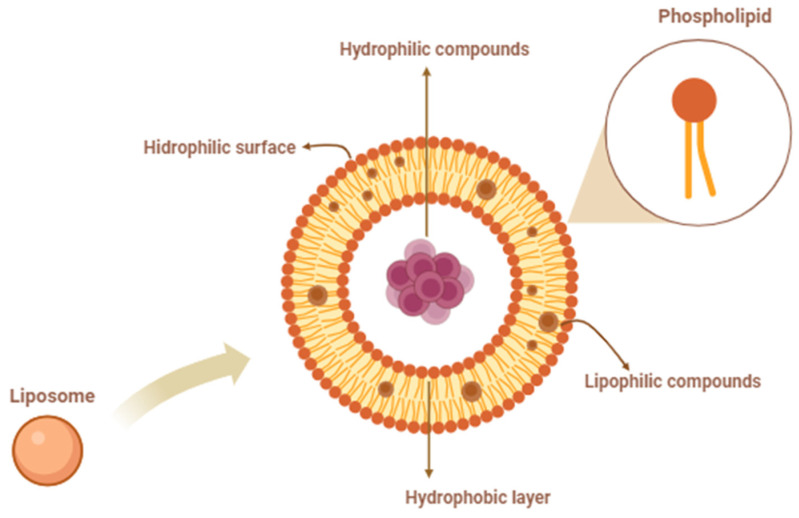
Liposome structure and bioactive compound incorporation.

Liposomes are microscopic structures formed from phospholipids, which are amphipathic molecules containing a hydrophilic head and hydrophobic tails. When phospholipids are dispersed in an aqueous environment, they spontaneously arrange themselves into bilayers, with the hydrophobic tails facing inward and the hydrophilic heads facing outward [31]. This arrangement creates a hydrophobic membrane and a hydrophilic exterior, allowing liposomes to encapsulate a variety of compounds (Figure 2). The structural versatility of liposomes is illustrated by their ability to accommodate both hydrophilic and lipophilic substances [11,32,33]. Hydrophilic compounds can be enclosed within the aqueous core of the liposome, while lipophilic compounds, like carotenoids, are integrated into the hydrophobic bilayer [6,34]. This dual capacity makes liposomes an ideal vehicle for delivering a wide range of bioactive molecules [16,35].

The primary function of liposomes in drug delivery is to provide protection and enhance the bioavailability and therapeutic index of bioactive compounds. By encapsulating them within liposomes, it is possible to protect the bioactive ingredient from degradation, enhance their absorption, and control their release. This is particularly valuable for drugs that have poor solubility and stability or that are rapidly metabolized in the body [36].

The study of liposomal formulations incorporating lipophilic extracts from sea buckthorn and water-soluble extracts from grape pomace revealed distinct encapsulation efficiencies, retention rates, and amounts of encapsulated bioactive compounds [37]. The formulations were prepared in two different dissolution media: double-distilled water and ethanol. The results, as presented in the Table 3, provide insights into the effectiveness of these liposomal systems.

The encapsulation efficiency (EE) measured the percentage of bioactive compounds successfully enclosed within the liposomal vesicles. The CDW sample exhibited the highest EE at 90.90%, indicating the superior encapsulation of carotenoids when water was used as the dissolution medium. This was followed closely by the PDW sample, with an EE of 89.59%. The CEt and PEt samples had slightly lower encapsulation efficiencies of 87.83% and 84.13%, respectively. The lower EE in ethanol might have been due to the solubility differences of the lipidic compounds in ethanol compared to water, affecting the formation and stability of the liposomal vesicles [18,19].

The retention rate (RR) indicated the stability of the encapsulated compounds within the liposomes after 4 weeks of storage. Similar to the encapsulation efficiency, the water-based formulations (CDW and PDW) demonstrated higher retention rates, with CDW at 86.74% and PDW at 84.79%. The ethanol-based formulations (CEt and PEt) showed lower retention rates, with CEt at 80.18% and PEt at 79.18%. The reduced retention in ethanol may be attributable to the potential interaction between ethanol and the lipid bilayer, possibly leading to a more permeable membrane and higher leakage of encapsulated compounds.

The amount of encapsulated bioactive compounds (EBA) reflected the actual quantity of bioactives trapped within the liposomes. The CDW sample contained the highest amount of encapsulated bioactive compounds at 83.74 μg, followed closely by CEt at 81.18 μg, PDW at 80.19 μg, and PEt at 78.98 μg. These results suggest that while both water and ethanol can be used as dissolution media, water tends to be slightly more effective in preserving higher amounts of encapsulated bioactives.

The findings indicate that liposomal formulations prepared in double-distilled water generally outperform those prepared in ethanol in terms of encapsulation efficiency, retention rate, and the amount of encapsulated bioactive compounds. The superior performance of water-based formulations could be attributed to the better stability and integrity of the liposomal bilayers in aqueous environments, which enhances the encapsulation and retention of bioactive compounds [38]. These insights are crucial for optimizing liposomal formulations for delivering carotenoids and polyphenols, suggesting that double-distilled water is a preferable medium for maximizing the encapsulation efficiency and stability of bioactive-loaded liposomes [36,39,40].

### 3.3. In Vitro Antioxidant Activity

This study investigated the antioxidant activity of liposomal formulations containing lipophilic extract from sea buckthorn and hydrophilic extract from grape pomace under simulated gastrointestinal conditions. The results revealed significant variations in antioxidant activity during gastric and intestinal digestion, emphasizing the influence of encapsulation and dissolution media on the stability and bioavailability of bioactive compounds [14].

Liposomal formulations were prepared using lipophilic extracts from sea buckthorn (CLE and LE) and hydrophilic extracts from grape pomace (PLE and PE). The antioxidant activity was measured during in vitro simulated gastric digestion (pH 1.8 ± 0.1) and, respectively, intestinal (pH 8.2 ± 0.1) digestion over 2 h.

**Figure 3:**
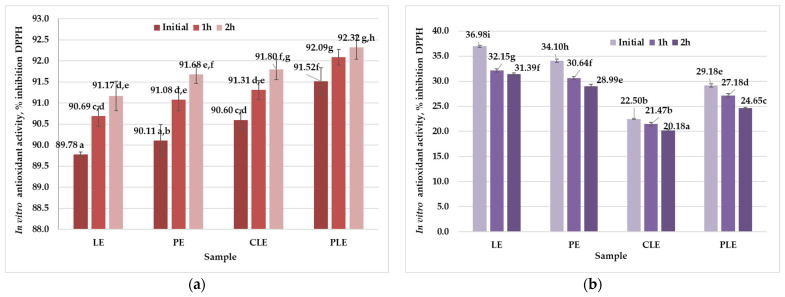
Antioxidant activity in vitro of liposomal formulations: (**a**) gastric digestion (pH = 1.8 ± 0.1); (**b**) intestinal digestion (pH = 8.2 ± 0.1). LE—lipophilic extract; PE—hydrophilic extract; CLE—carotenoid liposomal formulation; PLE—polyphenol liposomal formulation. The results are presented as the mean of three measurements ± standard deviation (SD). Different letters (a–i) designate statistically different results (*p* ≤ 0.05).

The gastric digestion (pH 1.8 ± 0.1) (Figure 3a) simulation demonstrated a progressive increase in antioxidant activity for all samples. Initial antioxidant activity for non-encapsulated lipophilic extract (LE) was 89.78% DPPH inhibition, increasing to around 91.17% DPPH inhibition after 2 h. Initial antioxidant activity for non-encapsulated hydrophilic extract (PE) was 90.11% DPPH inhibition, increasing to around 91.68% DPPH inhibition. Encapsulated carotenoids (CLE) and polyphenols (PLE) exhibited higher initial activities of around 90.60% DPPH inhibition and 91.52% DPPH inhibition, respectively, reaching approximately 91.80% DPPH inhibition and 92.32% DPPH inhibition after 2 h.

These findings confirm that the antioxidant activity of the carotenoid and polyphenol extracts was retained in the liposomal formulation. The increase in antioxidant activity during gastric digestion suggests that the acidic environment enhances the stability of bioactive compounds from liposomal formulations. The encapsulated hydrophilic extract rich in polyphenols exhibited higher antioxidant activity due to the better stability of the phenolic compounds in acidic conditions. The encapsulated formulations (CLE and PLE) showed superior performance compared to the non-encapsulated extracts (LE and PE), indicating that liposomal encapsulation protects incorporated compounds against acidic degradation. This highlights the ability of liposomes to maintain bioactive compound integrity in the stomach, potentially enhancing bioavailability [13,16,29]. Additionally, the incorporation of bioactive extracts (PLE and CLE) into the liposomal membrane with enhanced stability prevented bioactive extracts from interacting with the surroundings, thereby reducing the degradation of encapsulated extracts [41,42]. Pan et al. [41] obtained soybean liposomes dopped with astaxanthin and observed that the liposomes were stable in acidic pH (gastric phase), although a small increase in the size of the vesicles was recorded. When subjected to the intestinal phase (pH 7.4), a steep increase in the diameter of liposomes was observed, a phenomenon interpreted by the authors as the formation of mixed micelles. Moreover, a higher bioaccessibility of astaxanthin from liposomal formulations was observed compared to the free nonencapsulated form. A comprehensive review regarding the liposomal formulations and their food applications revealed that the structure of the liposomes was hardly affected in the pH conditions of the gastric phase (size decrease, aggregation) and the integrity of the membrane was maintained, thus protecting the entrapped material [42]. Contrarily, during the intestinal phase, liposome structure and integrity were strongly affected. An increase in the size of the particles was observed, accompanied by the destruction of the lipid bilayer due to the combined action of neutral/slightly alkaline pH and the enzymatic hydrolysis of phospholipids leading to the release of the encapsulated bioactive compounds. Several mechanisms were involved in the controlled release of molecules from liposomes, namely, diffusion, erosion, and swelling [42].

In contrast, during the simulated intestinal digestion (pH 8.2 ± 0.1) (Figure 3b), a decline in antioxidant activity over time for all samples was observed. Initial antioxidant activity for LE was about 35% DPPH inhibition, which decreased to 31% DPPH inhibition after 2 h. The antioxidant activity for PE was around 34% DPPH inhibition, which decreased to 28% DPPH inhibition. Both CLE and PLE exhibited initial activities of approximately 22% DPPH inhibition and 29% DPPH inhibition, which declined to around 20% DPPH inhibition and 24% DPPH inhibition after 2 h.

The decline in antioxidant activity during intestinal digestion suggests that the alkaline environment poses challenges both for the stability of liposomal formulations and for a part of encapsulated bioactive compounds. The intact structure of most liposomes undergoes damage and their encapsulated bioactive compounds are released. The non-encapsulated extracts (LE and PE) retained higher activity compared to the encapsulated forms (CLE and PLE), indicating that liposomal structures might be less stable or more prone to degradation in the intestine. Despite the decrease, the encapsulated bioactives still retained significant antioxidant activity, indicating that liposomal encapsulation offers some degree of protection even in less favorable conditions [5,28].

The results obtained demonstrate that liposomal encapsulation effectively enhances the antioxidant activity of bioactive compounds during gastric digestion. The encapsulated carotenoids and polyphenols showed higher stability and retention in the acidic environment compared to non-encapsulated extracts. However, during intestinal digestion, all samples experienced a decrease in antioxidant activity, with non-encapsulated extracts retaining more activity. This suggests that while liposomal encapsulation provides benefits in acidic conditions, further optimization is necessary to enhance stability and retention in alkaline environments. These findings highlight the importance of selecting appropriate encapsulation techniques and dissolution media to maximize the bioavailability and effectiveness of liposomal formulations under varying digestive conditions.

## 4. Conclusions

Sea buckthorn and grape pomace represent rich sources of bioactive compounds with antioxidant properties, namely, carotenoids and polyphenols. Lipophilic and hydrophilic extracts from both sources could be successfully encapsulated in liposomal formulations with efficiencies higher than 80%. Water-based formulations provided higher encapsulation efficiency and stability of liposomes loaded with bioactive compounds.

Important antioxidant activity was attested during gastric digestion in vitro. The encapsulated formulations showed superior performance compared to the unencapsulated extract, highlighting the protective effect of liposomal encapsulation against acid degradation. However, a reduction in antioxidant activity was observed during intestinal digestion, with non-encapsulated extracts retaining higher activity, suggesting that liposomal structures slow the release of bioactive compounds in the intestine.

The in vitro results support the effectiveness of liposomal formulations in preserving and enhancing the antioxidant activities of sea buckthorn and grape pomace extracts. This research provides a foundation for the further development and application of liposomal delivery systems in functional foods and nutraceuticals, aiming to improve human health through enhanced bioactive compound stability and bioavailability.

## Figures and Tables

**Table 1:** The carotenoid content in the pulp of sea buckthorn (*Hippophae rhamnoides* L.).

Carotenoid Compound	Retention Time, min	Max Absorption, nm	Quantity, mg/100 g FW
Zeaxanthin	10.01	426, 450, 476	0.72 ± 0.02
Cis-*β*-carotene	69.14	424, 446, 472	0.20 ± 0.01
All-trans-*β*-carotene	73.57	421, 452, 478	0.79 ± 0.03
Lycopene	95.55	448, 471, 503	0.14 ± 0.01
*β*-cryptoxanthin/zeaxanthin/mutatoxanthin esters (other than the ones below)	107.51		2.80 ± 0.04
	108.72		
	114.64		
	115.11		
	119.83		
Lutein dipalmitate	121.99	421, 446, 474	0.54 ± 0.05
Zeaxanthin dipalmitate	124.06	427, 450, 476	4.53 ± 0.12
Zeaxanthin palmitate-stearate	133.44	427, 450, 477	0.47 ± 0.06

The results are presented as the mean of three measurements ± standard deviation (SD). FW—fresh weight.

**Table 2:** Phenolic compound profiles in grape pomace extract.

Phenolic Compounds	Retention Time, min	Max Absorption, nm	Subclass	Quantity, mg/100 g DW
3-Hydroxybenzoic acid	2.99	270	Hydroxybenzoic acid	7.23 ± 0.15
2-Hydroxybenzoic acid	3.32	270	Hydroxybenzoic acid	7.08 ± 0.21
Gallic acid-gallate	5.94	270	Hydroxybenzoic acid	9.66 ± 0.08
Kaempferol-rutinoside	10.54	260, 340	Flavonol	1.05 ± 0.01
Prodelphinidin dimer B9	10.81	280	Flavanol	8.72 ± 0.15
Procyanidin dimmer B3	11.34	280	Flavanol	16.66 ± 0.18
Kaempferol-acetyl-glucoside	11.42	270, 340	Flavonol	2.10 ± 0.04
Peonidin-glucoside	11.78	280, 518	Anthocyanin	0.47 ± 0.01
Procyanidin dimmer B1	11.96	280	Flavanol	11.80 ± 0.21
Malvidin-glucoside	12.01	280, 528	Anthocyanin	0.84 ± 0.02
Catechin	12.43	280	Flavanol	20.47 ± 0.31
Caffeic acid-glucoside	12.91	332	Hydroxycinnamic acid	5.69 ± 0.05
3,4-Dicaffeoylquinic acid	13.43	332	Hydroxycinnamic acid	4.55 ± 0.08
Epicatechin	13.72	280	Flavanol	47.67 ± 0.33
Malvidin-glucoside-pyruvic acid	13.95	280, 530	Anthocyanin	1.06 ± 0.08
Dephinidin-p-coumaroyl-glucoside	14.17	280, 529	Anthocyanin	0.94 ± 0.02
Malvidin-acetyl-glucoside	14.52	280, 530	Anthocyanin	0.98 ± 0.01
Procyanidin dimmer B2	14.61	280	Flavanol	18.39 ± 0.19
Petunidin-p-coumaroyl-glucoside	14.77	280, 528	Anthocyanin	1.65 ± 0.03
Peonidin-p-coumaroyl-glucoside	15.17	280, 523	Anthocyanin	1.06 ± 0.01
Malvidin-caffeoyl-glucoside	15.62	280, 530	Anthocyanin	1.41 ± 0.02
Quercetin-glucoside	15.98	360, 250	Flavonol	3.24 ± 0.01
Malvidin-p-coumaroyl-glucoside	16.35	280, 530	Anthocyanin	0.90 ± 0.02
Quercetin-glucuronide	16.67	360, 250	Flavonol	3.63 ± 0.08
Isorhamnetin-glucoside	17.07	360, 260	Flavonol	2.88 ± 0.02
Quercetin-acetyl-glucoside	18.48	360, 250	Flavonol	1.07 ± 0.03
Quercetin	21.36	360, 250	Flavonol	0.86 ± 0.01

The results are presented as the mean of three measurements ± standard deviation (SD).

**Table 3:** Encapsulation efficiency, retention rate, and encapsulated bioactive compound amount of liposomal formulations.

Sample	Encapsulation Efficiency, %	Retention Rate after 4 Weeks of Storage, %	Encapsulated Bioactive Compound Amount, μg
CDW	90.90 ± 0.65 ^d^	86.74 ± 0.18 ^d^	83.74 ± 0.26 ^d^
CEt	87.83 ± 0.54 ^b^	80.18 ± 0.67 ^b^	81.18 ± 0.24 ^c^
PDW	89.59 ± 0.51 ^c^	84.79 ± 0.32 ^c^	80.19 ± 0.38 ^b^
PEt	84.13 ± 0.29 ^a^	79.18 ± 0.21 ^a^	78.98 ± 0.12 ^a^

CDW—carotenoid/distilled water liposomal formulation; CEt—carotenoid/ethanol liposomal formulation; PDW—polyphenols/distilled water liposomal formulation; PEt—polyphenols/ethanol liposomal formulation. The results are presented as the mean of three measurements ± standard deviation (SD). Different letters (^a–d^) designate statistically different results (*p* ≤ 0.05).

## Data Availability

The original contributions presented in this study are included in the article/Supplementary Material; further inquiries can be directed to the corresponding author.

## Supplementary Materials

The following supporting information can be downloaded at https://www.mdpi.com/article/10.3390/foods13162478/s1: Figure S1. The grape phenolic chromatogram for 280 nm; Figure S2. The grape phenolic chromatogram for 340 nm; Figure S3. The grape phenolic chromatogram for 520 nm.

## References

[1] Liu P., Chen G., Zhang J. (2022). A Review of Liposomes as a Drug Delivery System: Current Status of Approved Products, Regulatory Environments, and Future Perspectives. Molecules.

[2] Gómez-Alonso S., Mancebo-Campos V., Desamparados Salvador M., Fregapane G. (2004). Oxidation Kinetics in Olive Oil Triacylglycerols under Accelerated Shelf-Life Testing (25–75 °C). Eur. J. Lipid Sci. Technol..

[3] Kaur D., Wani A.A., Singh D.P., Sogi D.S. (2011). Shelf Life Enhancement of Butter, Ice-Cream, and Mayonnaise by Addition of Lycopene. Int. J. Food Prop..

[4] Berger M. (2005). Can Oxidative Damage Be Treated Nutritionally?. Clin. Nutr..

[5] Li Y., Tan X., Liu X., Liu L., Fang Y., Rao R., Ren Y., Yang X., Liu W. (2020). Enhanced Anticancer Effect of Doxorubicin by TPGS-Coated Liposomes with Bcl-2 siRNA-Corona for Dual Suppression of Drug Resistance. Asian J. Pharm. Sci..

[6] Chai C., Park J. (2024). Food Liposomes: Structures, Components, Preparations, and Applications. Food Chem..

[7] Eidhin D.N., Burke J., O’Beirne D. (2003). Oxidative Stability of Ω3-Rich Camelina Oil and Camelina Oil-Based Spread Compared with Plant and Fish Oils and Sunflower Spread. J. Food Sci..

[8] Song F., Chen J., Zheng A., Tian S. (2022). Effect of Sterols on Liposomes: Membrane Characteristics and Physicochemical Changes during Storage. LWT.

[9] Jahanfar S., Gahavami M., Khosravi-Darani K., Jahadi M., Mozafari M.R. (2021). Entrapment of Rosemary Extract by Liposomes Formulated by Mozafari Method: Physicochemical Characterization and Optimization. Heliyon.

[10] Jara-Quijada E., Pérez-Won M., Tabilo-Munizaga G., Lemus-Mondaca R., González-Cavieres L., Palma-Acevedo A., Herrera-Lavados C. (2024). Liposomes Loaded with Green Tea Polyphenols—Optimization, Characterization, and Release Kinetics under Conventional Heating and Pulsed Electric Fields. Food Bioprocess Technol..

[11] Liu X., Wang P., Zou Y.-X., Luo Z.-G., Tamer T.M. (2020). Co-Encapsulation of Vitamin C and β-Carotene in Liposomes: Storage Stability, Antioxidant Activity, and In Vitro Gastrointestinal Digestion. Food Res. Int..

[12] Akbarzadeh A., Rezaei-Sadabady R., Davaran S., Joo S.W., Zarghami N., Hanifehpour Y., Samiei M., Kouhi M., Nejati-Koshki K. (2013). Liposome: Classification, Preparation, and Applications. Nanoscale Res. Lett..

[13] Liu Y., Liu D., Zhu L., Gan Q., Le X. (2015). Temperature-Dependent Structure Stability and In Vitro Release of Chitosan-Coated Curcumin Liposome. Food Res. Int..

[14] Gibis M., Ruedt C., Weiss J. (2016). In Vitro Release of Grape-Seed Polyphenols Encapsulated from Uncoated and Chitosan-Coated Liposomes. Food Res. Int..

[15] da Silva Haas I.C., Toaldo I.M., Gomes T.M., Luna A.S., de Gois J.S., Bordignon-Luiz M.T. (2019). Polyphenolic Profile, Macro- and Microelements in Bioaccessible Fractions of Grape Juice Sediment Using in Vitro Gastrointestinal Simulation. Food Biosci..

[16] Xu X., Tian M., Deng L., Jiang H., Han J., Zhen C., Huang L., Liu W. (2023). Structural Degradation and Uptake of Resveratrol-Encapsulated Liposomes Using an In Vitro Digestion Combined with Caco-2 Cell Absorption Model. Food Chem..

[17] Witika B.A., Makoni P.A., Matafwali S.K., Mweetwa L.L., Shandele G.C., Walker R.B. (2021). Enhancement of Biological and Pharmacological Properties of an Encapsulated Polyphenol: Curcumin. Molecules.

[18] Srinivasan V., Chavan S., Jain U., Tarwadi K., Pudake R.N., Chauhan N., Kole C. (2019). Liposomes for Nanodelivery Systems in Food Products. Nanoscience for Sustainable Agriculture.

[19] Rudzińska M., Grygier A., Knight G., Kmiecik D. (2024). Liposomes as Carriers of Bioactive Compounds in Human Nutrition. Foods.

[20] Ghendov-Mosanu A., Popovici V., Constantinescu (Pop) C.G., Deseatnicova O., Siminiuc R., Subotin I., Druta R., Pintea A., Socaciu C., Sturza R. (2023). Stabilization of Sunflower Oil with Biologically Active Compounds from Berries. Molecules.

[21] Ghendov-Mosanu A., Cristea E., Patras A., Sturza R., Niculaua M. (2020). Rose Hips, a Valuable Source of Antioxidants to Improve Gingerbread Characteristics. Molecules.

[22] Ghendov-Moşanu A., Sturza R., Opriş O., Lung I., Popescu L., Popovici V., Soran M.-L., Patraş A. (2020). Effect of Lipophilic Sea Buckthorn Extract on Cream Cheese Properties. J. Food Sci. Technol..

[23] Morar I.M., Stefan R., Dan C., Sestras R.E., Truta P., Medeleanu M., Ranga F., Sestras P., Truta A.M., Sestras A.F. (2024). FT-IR and HPLC Analysis of Silver Fir (*Abies alba* Mill.) Bark Compounds from Different Geographical Provenances. Heliyon.

[24] Tudor C., Bohn T., Iddir M., Dulf F.V., Focşan M., Rugină D.O., Pintea A. (2019). Sea Buckthorn Oil as a Valuable Source of Bioaccessible Xanthophylls. Nutrients.

[25] Brand-Williams W., Cuvelier M.E., Berset C. (1995). Use of a Free Radical Method to Evaluate Antioxidant Activity. LWT—Food Sci. Technol..

[26] Popovici V. (2018). Evaluation of the Oxidative Stability of Sea Buckthorn (*Hippophae Rhamnoides* L.) Lipophilic Extract. J. Eng. Sci..

[27] Rasti B., Jinap S., Mozafari M.R., Yazid A.M. (2012). Comparative Study of the Oxidative and Physical Stability of Liposomal and Nanoliposomal Polyunsaturated Fatty Acids Prepared with Conventional and Mozafari Methods. Food Chem..

[28] Tan C., Xue J., Lou X., Abbas S., Guan Y., Feng B., Zhang X., Xia S. (2014). Liposomes as Delivery Systems for Carotenoids: Comparative Studies of Loading Ability, Storage Stability and In Vitro Release. Food Funct..

[29] Pavan V., Sancho R.A.S., Pastore G.M. (2014). The Effect of in Vitro Digestion on the Antioxidant Activity of Fruit Extracts (*Carica papaya*, *Artocarpus heterophillus* and *Annona marcgravii*). LWT-Food Sci. Technol..

[30] Dai J., Mumper R.J. (2010). Plant Phenolics: Extraction, Analysis and Their Antioxidant and Anticancer Properties. Molecules.

[31] Mattioli R., Francioso A., Mosca L., Silva P. (2020). Anthocyanins: A Comprehensive Review of Their Chemical Properties and Health Effects on Cardiovascular and Neurodegenerative Diseases. Molecules.

[32] Carvajal A.K., Rustad T., Mozuraityte R., Storrø I. (2009). Kinetic Studies of Lipid Oxidation Induced by Hemoglobin Measured by Consumption of Dissolved Oxygen in a Liposome Model System. J. Agric. Food Chem..

[33] Andra V.V.S.N.L., Pammi S.V.N., Bhatraju L.V.K.P., Ruddaraju L.K. (2022). A Comprehensive Review on Novel Liposomal Methodologies, Commercial Formulations, Clinical Trials and Patents. BioNanoScience.

[34] Islam Shishir M.R., Karim N., Gowd V., Zheng X., Chen W. (2019). Liposomal Delivery of Natural Product: A Promising Approach in Health Research. Trends Food Sci. Technol..

[35] Toh M.-R., Chiu G.N.C. (2013). Liposomes as Sterile Preparations and Limitations of Sterilisation Techniques in Liposomal Manufacturing. Asian J. Pharm. Sci..

[36] Sercombe L., Veerati T., Moheimani F., Wu S.Y., Sood A.K., Hua S. (2015). Advances and Challenges of Liposome Assisted Drug Delivery. Front. Pharmacol..

[37] Popovici V., Pintea A., Boldianu A., Mereuta I., Caraus V., Cicalchin S., Covaci E., Ghendov-Mosanu, Sturza R. (2024). Liposomal Encapsulated Extract with Biologically Active Lipophilic or Hydrophilic Substances, Which Possess Antioxidant and Immunostimulatory Activity, and Method for Obtaining It, AGEPI, Patent Application. Entry.

[38] Encina C., Vergara C., Giménez B., Oyarzún-Ampuero F., Robert P. (2016). Conventional Spray-Drying and Future Trends for the Microencapsulation of Fish Oil. Trends Food Sci. Technol..

[39] Šeregelj V., Tumbas Šaponjac V., Lević S., Kalušević A., Ćetković G., Čanadanović-Brunet J., Nedović V., Stajčić S., Vulić J., Vidaković A. (2019). Application of Encapsulated Natural Bioactive Compounds from Red Pepper Waste in Yogurt. J. Microencapsul..

[40] Oluwatuyi M., Kaatz G., Gibbons S. (2004). Antibacterial and Resistance Modifying Activity of of *Rosmarinus officinalis*. Phytochemistry.

[41] Pan L., Meng H., Li J., Liu Z., Zhang D., Liu Z., Zhao Q., Xu F. (2024). Enhancement of Astaxanthin Bioaccessibility by Encapsulation in Liposomes: An In Vitro Study. Molecules.

[42] Liu W., Hou Y., Jin Y., Wang Y., Xu X., Han J. (2020). Research Progress on Liposomes: Application in Food, Digestion Behavior and Absorption Mechanism. Trends Food Sci. Technol..

