# Supplementary figures and images for: In Vitro Antioxidant Activity of Liposomal Formulations of Sea Buckthorn and Grape Pomace
Source: Foods. 2024 Aug 7;13(16):2478. doi: 10.3390/foods13162478 (PMC11354042; doi:10.3390/foods13162478)

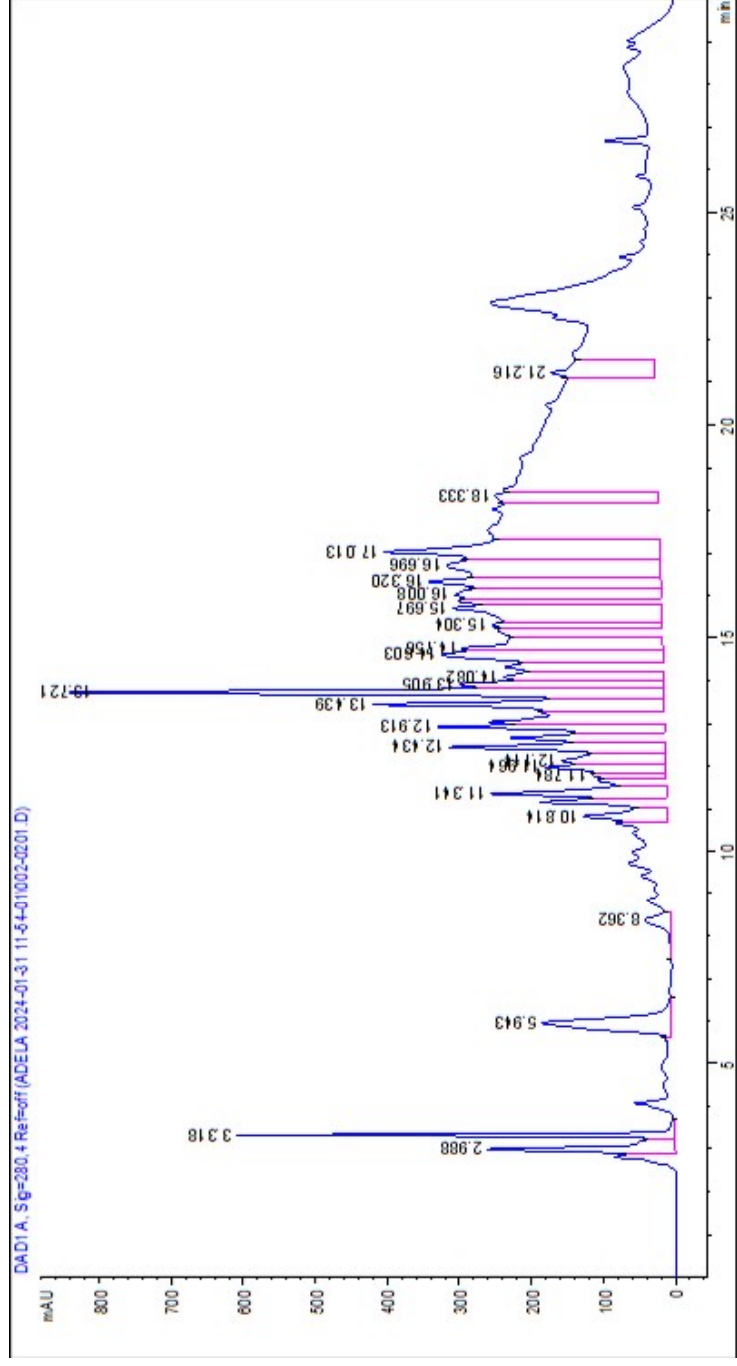

Supplement: Supplementary file 1 [file foods-13-02478-s001.zip › Figure S1. The grape phenolic chromatogram for 280 nm.pdf]

DAD15, Sig=340.4 Ref=off (ADELA 2024-01-31 11:54-01003-0201.D)

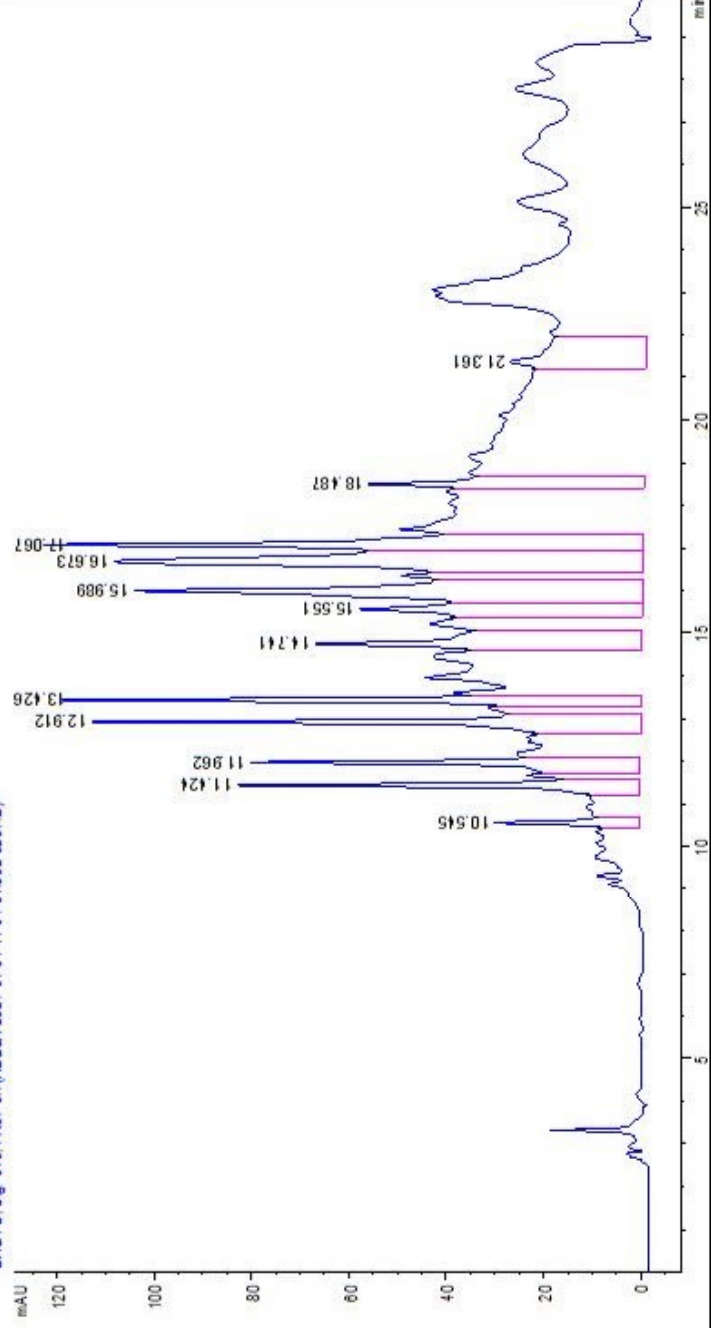

Supplement: Supplementary file 1 [file foods-13-02478-s001.zip › Figure S2. The grape phenolic chromatogram for 340 nm.pdf]

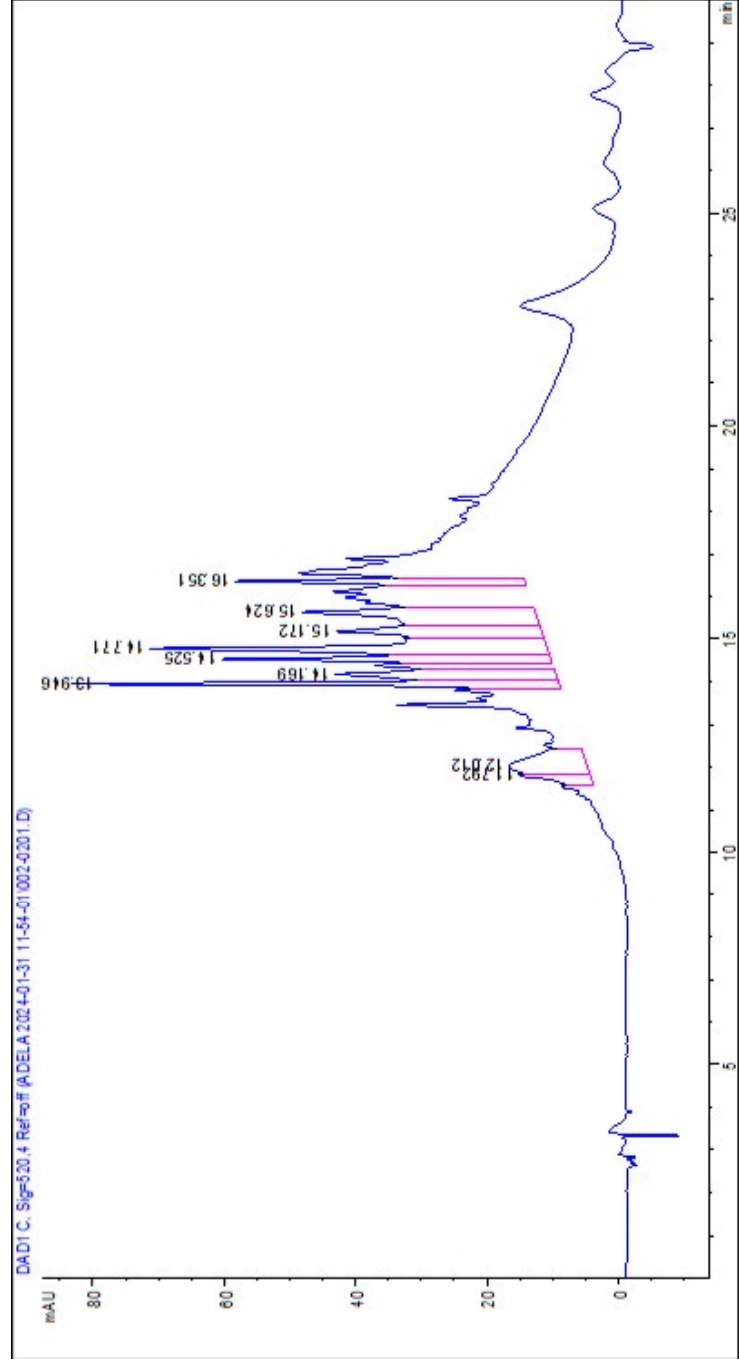

Supplement: Supplementary file 1 [file foods-13-02478-s001.zip › Figure S3. The grape phenolic chromatogram for 520 nm.pdf]
